# Optimizing vitiligo diagnosis with ResNet and Swin transformer deep learning models: a study on performance and interpretability

**DOI:** 10.1038/s41598-024-59436-2

**Published:** 2024-04-21

**Authors:** Fan Zhong, Kaiqiao He, Mengqi Ji, Jianru Chen, Tianwen Gao, Shuli Li, Junpeng Zhang, Chunying Li

**Affiliations:** 1 College of Electrical Engineering, Sichuan University, Chengdu, China; 2 Department of Dermatology, Xijing Hospital, Fourth Military Medical University, Xi’an, China

**Keywords:** Vitiligo, Swin transformer, Dermoscopic images, Class activation mapping, Vitiligo, Preclinical research

## Abstract

Vitiligo is a hypopigmented skin disease characterized by the loss of melanin. The progressive nature and widespread incidence of vitiligo necessitate timely and accurate detection. Usually, a single diagnostic test often falls short of providing definitive confirmation of the condition, necessitating the assessment by dermatologists who specialize in vitiligo. However, the current scarcity of such specialized medical professionals presents a significant challenge. To mitigate this issue and enhance diagnostic accuracy, it is essential to build deep learning models that can support and expedite the detection process. This study endeavors to establish a deep learning framework to enhance the diagnostic accuracy of vitiligo. To this end, a comparative analysis of five models including ResNet (ResNet34, ResNet50, and ResNet101 models) and Swin Transformer series (Swin Transformer Base, and Swin Transformer Large models), were conducted under the uniform condition to identify the model with superior classification capabilities. Moreover, the study sought to augment the interpretability of these models by selecting one that not only provides accurate diagnostic outcomes but also offers visual cues highlighting the regions pertinent to vitiligo. The empirical findings reveal that the Swin Transformer Large model achieved the best performance in classification, whose AUC, accuracy, sensitivity, and specificity are 0.94, 93.82%, 94.02%, and 93.5%, respectively. In terms of interpretability, the highlighted regions in the class activation map correspond to the lesion regions of the vitiligo images, which shows that it effectively indicates the specific category regions associated with the decision-making of dermatological diagnosis. Additionally, the visualization of feature maps generated in the middle layer of the deep learning model provides insights into the internal mechanisms of the model, which is valuable for improving the interpretability of the model, tuning performance, and enhancing clinical applicability. The outcomes of this study underscore the significant potential of deep learning models to revolutionize medical diagnosis by improving diagnostic accuracy and operational efficiency. The research highlights the necessity for ongoing exploration in this domain to fully leverage the capabilities of deep learning technologies in medical diagnostics.

## Introduction

Skin diseases present a substantial healthcare challenge worldwide, with vitiligo standing out as one of the prevalent conditions. It is a dermatological condition characterized by the progressive loss of melanocytes, resulting in depigmentation of the skin. The progressive nature of vitiligo can profoundly impact patients' physical and psychological well-being^1^. Consequently, prompt and accurate diagnosis is pivotal for facilitating effective treatment interventions.

Various diagnostic methods, including dermoscopy, wood lamp examination, skin CT scans, and skin biopsies, are utilized in the diagnosis of skin conditions. Dermoscopy, in particular, affords comprehensive insights into the status of melanocytes and the distinctive characteristics of vitiligo patches^2,3^. By recognizing the pigment cell loss or reduction and identifying structural changes within areas of depigmentation, it contributes to the diagnosis of vitiligo. Apart from this, skin biopsy and histological examination can also be employed to evaluate the condition of pigment cells and confirm the presence of vitiligo. Nevertheless, since skin biopsy is invasive, it is not used for routine diagnosis. Typically, the clinical diagnosis of vitiligo relies on a combination of physical examination, dermoscopy, and wood lamp examination. There is no single diagnostic test that conclusively confirms vitiligo, which requires the involvement of a dermatologist with expertise in vitiligo.

Unfortunately, there is a shortage of dermatologists and an unequal distribution of medical resources. In some remote areas, even non-dermatologists have to undertake the diagnosis and treatment of vitiligo due to medical resource constraints, despite their limited knowledge and training in this field. Although dermatology textbooks can be used as reference material, accurate identification and diagnosis is still the main challenge for these laypersons. As a result, rates of misdiagnosis and underdiagnosis remain high, and diagnostic accuracy ranges from 24 to 70%^4–7^. Therefore, the development of accurate and efficient Artificial Intelligence (AI) -assisted diagnostic tools is crucial for analyzing vitiligo dermoscopy images. The AI-assisted diagnostic tools hold the potential to furnish dermatologists with precise classification results, thereby contributing to the accuracy of vitiligo diagnosis. This technology also serves to mitigate potential errors stemming from limited expertise, especially in the context of non-dermatologists.

The major attention of AI-assisted diagnostic tools is to achieve more accurate classification of medical images. As early as 1959, AI-assisted diagnostic tools have been used in medicine. Initially, some traditional machine learning models are widely used for dermatological classification problems, such as Support Vector Machines (SVM)^8^, K-nearest neighbor (KNN)^9^, and Naive Bayes^10^. Unfortunately, these machine learning models are heavily reliant on the quality of manual feature extraction, which poses challenges in simultaneously achieving more precise classification results and lower system complexity. Furthermore, the utilization of hand-crafted features^11^ in these models significantly hampers both the performance and generalisability^12^ of the models when applied to dermoscopic images.

In contrast to traditional machine learning methods, deep learning has shown superior performance and has attracted more attention. Its effectiveness has been prominently demonstrated in various medical image-processing applications^13–15^. The adoption of automatic feature extraction has made it becoming more and more popular in the dermatological image classification field^16–18^. As early as 2017, deep learning architectures have been proposed and utilized in the ISIC 2017 Dermoscopy Image Segmentation Challenge for dermatological classification, segmentation, and detection tasks^19–21^. Notably, ResGANet has exhibited outstanding performance in medical image classification tasks in comparison to state-of-the-art backbone models^22^. Moreover, ResGANet has demonstrated the ability to enhance performance in medical image segmentation tasks by combining it with various segmentation networks. Therefore, deep learning-based methods can effectively overcome the limitations associated with traditional machine learning methods.

However, it is crucial to acknowledge the notable challenge known as the “black box” problem in deep learning methods. Despite the relative simplicity of the mathematical theory, the output mechanism is difficult to understand. In the field of medical image processing, the interpretability of classification results is crucial. However, the existence of the “black box” problem prevents physicians and researchers from understanding the logic and mechanisms of implementing these methods^23^. This lack of interpretability undermines the reliability of deep learning methods, thus limiting their use in clinical practice. To break through this constraint, visualization tools and techniques can be used to increase the transparency of the model and enhance interpretability. Several researchers have explored the application of weakly supervised semantic segmentation. This method utilizes image-level labeling information, such as identifying the presence or absence of a lesion, to infer the segmentation results of the lesion region. This method significantly improves the interpretability of medical image classification results^24–26^, since the segmented lesion regions correspond to the visual observations of the physician. Generally, this methodology is acknowledged for its capacity to obviate the requirement for manual processing of segmentation masks employed as training labels, resulting in substantial time and effort conservation^27^. Nevertheless, the delineation of ground truth remains imperative during the training of the segmentation network, necessitating manual creation by domain experts.

This paper focuses on a AI-assisted diagnostic system based on deep learning methods, using dermoscopic images for the detection and assessment of vitiligo. The objective of the system is to provide a diagnostic outcome of vitiligo and a visual diagnostic report that highlights potential areas associated with the disease. For this purpose, a set of networks has been trained, and the top five deep learning models with the most favorable results were ultimately selected for comparison. These models belong to the Residual Network (ResNet) and Swin Transformer network series. The results reveal that the Swin Transformer, which is a series of image classification models based on Transformer architecture, attains the highest accuracy in vitiligo classification. This model effectively handles global dependencies in images through hierarchical attention mechanisms and cross-stage connectivity mechanisms. Diverging from conventional semantic segmentation techniques prevalent in medical image processing and weakly supervised semantic segmentation methods, the deep learning models used in this paper were exclusively trained by using disease category labels. With the overall training process, the deep learning-based method achieves unsupervised learning for the regions of interest(ROI).

## Materials and methods

### A. Materials

The study obtained approval from the Ethics Committee at the Fourth Military Medical University, following the principles outlined in the Declaration of Helsinki. The dermoscopic image dataset utilized for both model training and testing was sourced from the Department of Dermatology at Xijing Hospital, affiliated with the Fourth Military Medical University. In accordance with confidentiality regulations and exclusions, no additional clinical data from patients were collected. The dataset consisted of a total of 4320 dermoscopic images, representing eight distinct hypopigmented skin diseases. Among these, 2678 images were specifically associated with vitiligo, while the remaining 1642 images represented seven other pigmented skin diseases distinct from vitiligo. These seven hypopigmented dermatoses were identified as pityriasis alba, pityriasis versicolor, marshall white syndrome, anemic nevus, idiopathic guttate hypomelanosis, amelanotic nevus, and hypomelanosis of Ito. Considering that the primary objective of this study was to distinguish vitiligo through dermoscopic images, which constitutes a dichotomous problem, the other seven pigmented dermatoses were collectively grouped. To enhance the assessment of the model, the dataset was divided into training and test sets in a 7:3 ratio. Within the training dataset, there are 1875 images depicting cases of vitiligo and 1150 images of non-vitiligo cases. As for the test dataset, it comprises 803 images representing vitiligo cases and 492 images non-vitiligo cases. All images were captured in the RGB color space and resized to a standardized dimension of 1280*960 pixels for both model training and testing. Considering the bias of the dataset on vitiligo conditions, an attention mechanism is introduced in the model to focus more on the features of non-vitiligo images, thus balancing the bias in the training process.

### B. Data preprocessing and data enhancement

In order to improve the richness of the data during the subsequent training of the network, and thus improve the anti-interference ability and generalization of the model, conventional preprocessing methods such as filtering, segmentation, hair removal, etc., are not applied to the raw data. Considering that the lighting conditions may be different at the time of data acquisition, color constancy processing is used in this study to attenuate this effect.

The goal of color constancy is to transform the image acquired under an unknown light source so that the processed image is close to the image acquired under a standard light source. Typically, color constancy processing can be accomplished in two separate steps. First, the estimation of the light source in RGB space is accomplished. The estimated light source is then used to transform the image to minimize the effect of the light source. The Shades of Gray method, which is the most commonly used method for dermatoscopic image processing, employs Minkowski's paradigm for light source estimation. It uses Minkowski's paradigm to estimate the light source. The value of P can be changed automatically, and when P = 1, the method degenerates to the GrayWorld algorithm. When P = ∞, the equation is equivalent to finding the maximum value of f(x), which is equivalent to the MaxRGB method. In this study, the value of P is set as 6. The steps of the calculation and the Minkowski paradigm used are shown below:

$$(\frac{{\int {(f(X)^{P} dX)} }}{{\int {dX} }})^{\frac{1}{p}} = ke$$

(1) Substitute the data of each channel into the Minkowski paradigm to find the Min distance of each channel; (2) Substitute the data of the whole image into the Minkowski paradigm to find the Min distance of the whole; (3) Calculate the ratio of the correction according to the distance of the whole and the distance of each channel; (4) Perform the correction of the ratio of each channel, and check whether there is any value exceeding the threshold value, and set it as 255 for the ones exceeding the threshold value of 255. All images are preprocessed to replace the original images, and by reading the label file, the images can be mapped to the corresponding dermatologic category.

**Figure 1:**
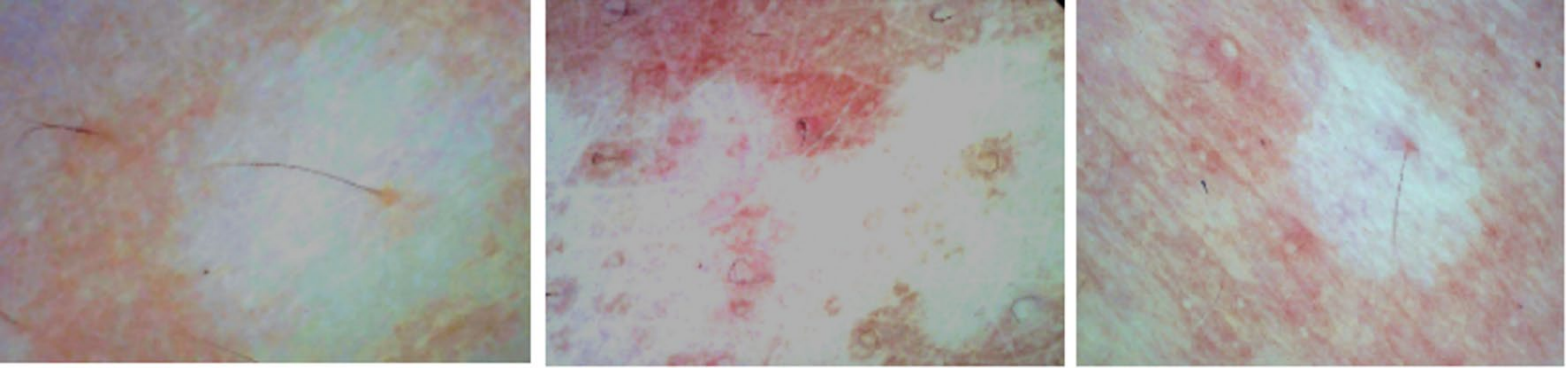
Three sample images following data augmentation procedures.

After the preprocessing was completed, to further improve the model performance and results, we used a data augmentation technique in each training iteration to make some minor changes to the data as a preliminary step before batch sampling. This data augmentation strategy employed in this study encompassed four distinct methods, namely random rotation, random brightness, random contrast, and random saturation. Additionally, to maintain the original image dimensions, any empty spaces resulting from the data augmentation procedures were filled with black pixels. Visual illustrations of the input images following the application of the data augmentation procedures are presented in Fig. 1.

### C. Overview of the ResNet networks

**Figure 2:**
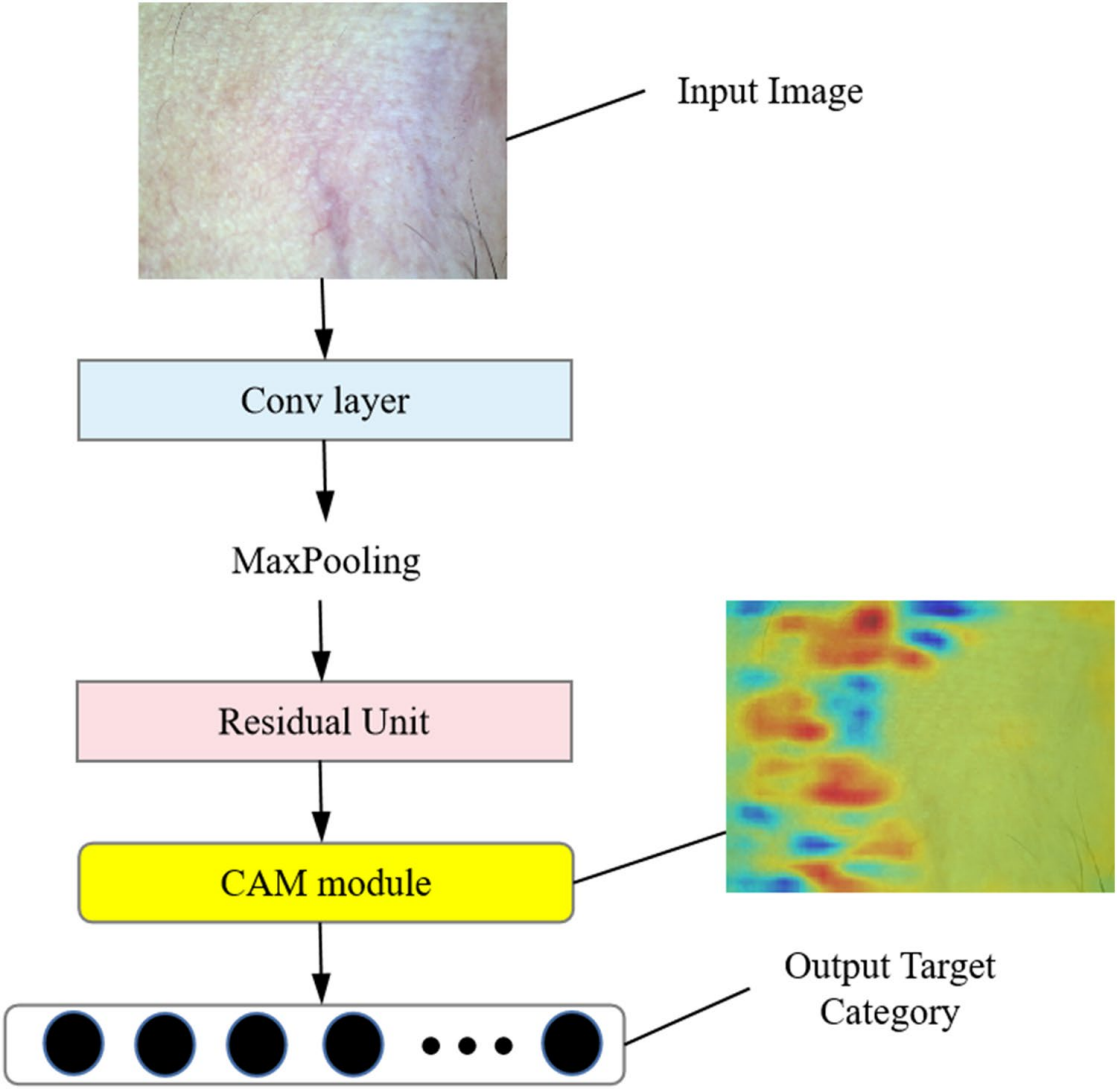
A brief overview of the ResNet framework with the addition of the CAM module.

The ResNet network architecture includes preprocessing layers, and residual units, as well as a fully connected layer and a Softmax layer. The key innovation of ResNet resides in its incorporation of residual connectivity, which effectively addresses the challenges of gradient vanishing and exploding encountered during the training of deep networks, by establishing direct interconnections between layers. In conventional deep networks, the increase in the number of layers results in a degradation of the gradient, thereby creating challenges in network training. In order to enhance the network's interpretability and visual comprehension, a Class activation mapping (CAM) module is incorporated into the ResNet architecture (as depicted in Fig. 2). The CAM module plays a crucial role in comprehending how the network allocates attention to different categories and identifies significant image regions. This network architecture facilitates both image classification and the generation of category activation maps simultaneously. This capability allows the network not only to predict the input image's category but also to provide visual interpretations of the classification outcomes. To enhance the analysis and comprehension of the feature extraction process within the network, as well as to support research and applications in feature visualization and analysis, the hook technique is utilized in conjunction with ResNet. This technique involves the extraction of feature output maps from intermediate layers of the neural network by registering hook functions during the forward propagation process. Through the implementation of the hook technique, the feature outputs of the middle layer can be acquired, thus enabling thorough exploration and analysis of the network's feature extraction capabilities.

### D. Overview of the Swin transformer networks

Swin Transformer^28^ has been proposed by Microsoft Research in 2021 as a deep neural network model based on the Transformer architecture. Its primary objective is to extend the application of the Transformer model into the realm of image processing by incorporating a layered window attention mechanism. In contrast to traditional Transformers, the Swin Transformer employs a Shifted-Window-based Multi-head Self-Attention (SW-MSA) module. This module is instrumental in modeling images at various granularities, contributing to the model’s enhanced performance in capturing diverse features within the image data. This design allows the Swin Transformer to effectively process large-sized images while maintaining computational complexity low during inference. Furthermore, the Swin Transformer consists of multiple Transformer layers, forming a deep network structure. To improve the model's local perception, an interaction layer is introduced. This layer facilitates the information exchange and interaction between different windows.

In comparison to the Vision Transformer (VIT), the Swin Transformer introduces a hierarchical structure reminiscent of a convolutional neural network (CNN), marking a significant enhancement over VIT. Another notable improvement involves replacing the multi-headed self-attention (MSA) module with a SW-MSA module. Summarizing these two improvements as hierarchical feature mapping and SW-MSA. Hierarchical feature mapping requires the work of downsampling, which is commonly used in image recognition before pooling operations. Instead of traditional pooling, the Swin Transformer employs Patch Merging for downsampling, reducing both height (H) and width (W) by half, and channels (C) by four times. These modifications effectively address VIT's challenges in fine-grained tasks and excessive complexity, respectively. In terms of the skeleton, the structural skeleton of VIT is still used for the design and continues to process input image data using the same patched.

In terms of scale, the Swin Transformer provides various model specifications tailored to different tasks and resource constraints. The three main types of Swin Transformers are Base, Large, and Tiny, and the two specific scales used in this study are Base and Large.

### E. Swin transformer attention module

The attention mechanism serves as a computational model designed to identify and assign weights to relevant elements within a sequence or set. This process involves computing an attention weight by learning the relationship between a query (Q), a key (K), and a value (V). Subsequently, this weight is applied to the corresponding value to produce a weighted representation. In practical applications, the Self-Attention mechanism has found extensive use. The MSA is an extended version of the self-attentive mechanism, aiming to enhance the representation capability of the model. It applies the attention mechanism to multiple attention heads (i.e., multiple Q, K, and V). Each attention head within the MSA mechanism is capable of learning distinct weights, allowing it to focus on different information within the input sequence. Finally, the outputs from these multiple attention heads are combined or aggregated to produce the final representation vector. MSA is extensively employed in Transformer models to capture global dependencies present in the input sequence. And the Swin Transformer mainly contains Window-Based Multiple Self-Attention (W-MSA) and SW-MSA.

**Figure 3:**
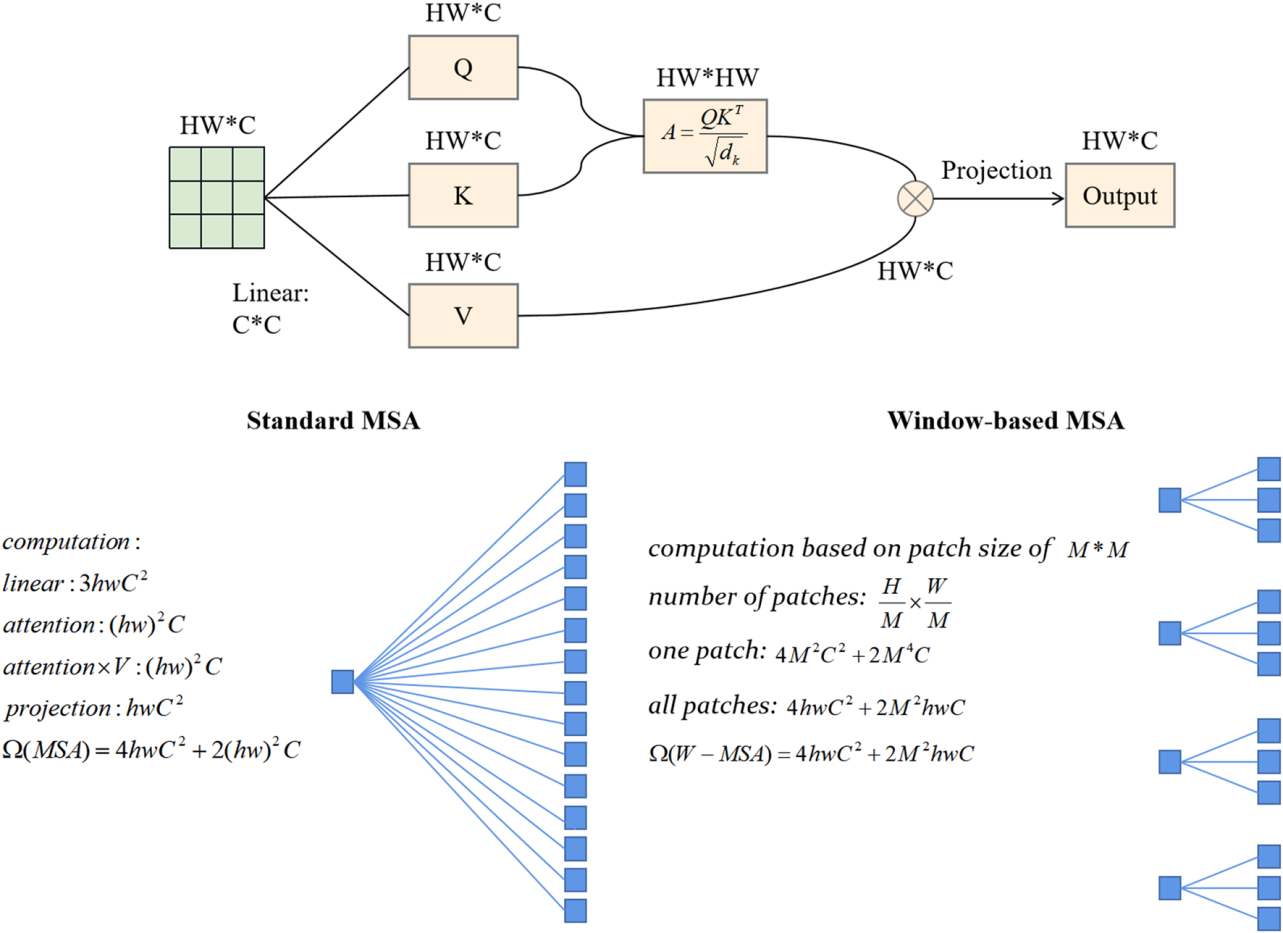
Computational complexity of self-attention mechanisms for MSA and W-MSA.

The input features are partitioned into multiple equally sized, non-overlapping windows in W-MSA, each treated as an individual attention head. In this way, the number of windows directly corresponds to the number of attention heads. For each window, W-MSA utilizes self-attention to compute dependencies between various positions within the window. This involves calculating the similarity between Q and K (usually using dot product attention or scaled dot product attention). Consequently, the attention weights for different positions within the window are determined, and the aggregation of values within the window is weighted using these attentional weights. This process produces the feature representation within the window. Its main objective is to tackle the problem of excessive memory usage linked to VIT's Self-Attention mechanism. The computational complexity of the self-attention mechanism is illustrated in Fig. 3 for both MSA and W-MSA. W-MSA effectively reduces the complexity of MSA from $O(n^{{2}} )$ to $O(n)$, alleviating the memory footprint constraints associated with VIT. Nonetheless, W-MSA has its limitations, and confining attention to the window introduces the challenge of global attention loss. To address this problem, the Swin Transformer integrates the SW-MSA module following the W-MSA module. This window-shifting approach introduces essential inter-window connections, thereby improving the network's overall performance. The SW-MSA module splits the image into non-overlapping blocks and computes the attention of each block with the neighboring blocks, which enables the model to pay more attention to the local information of the image. In order to solve the problem of mismatched attention due to interactive movement between windows, a mask matrix is added. For each window, a mask matrix is designed separately, in which the mask matrix is assigned to -100 for the part that should not be computed, so that after subsequent Softmax computation, it will eventually become 0, which is equivalent to playing a filtering role. In addition, the SW-MSA module can establish long-distance dependencies between different locations through the multi-head self-attention mechanism, which helps to capture the correlation information between different locations in the image and improves the model's ability to perceive the global information of the image.

### F. Class activation mapping module

**Figure 4:**
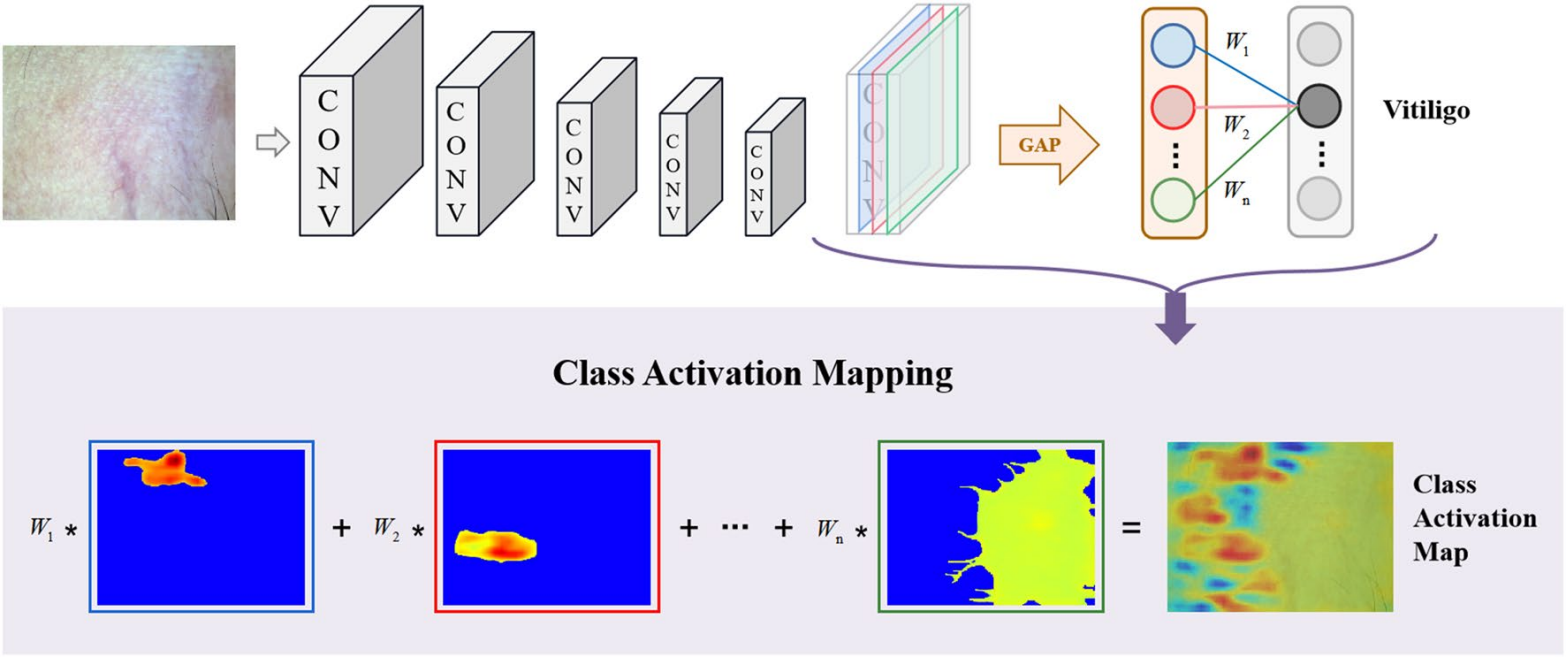
Graphical diagram of the CAM module mechanism.

To visualize the classification results, a CAM module is incorporated before the final output layer, comprising a global average pooling (GAP) layer and a dense layer. For instance, as depicted in Fig. 4, the CAM module takes the output feature map of the last residual module as input. It then applies GAP to the feature map, resulting in a fixed-length vector. This vector undergoes dot-product computation with the weights in the final fully connected layer, yielding activation values for each category. These activation values can be interpreted as the significance of the region associated with a particular category within the input image. By weighting and summing the input feature mapping values of the last layer of the residual unit, the CAM can be obtained. In the calculation, it is assumed that $f_{k} (x,y)$ represents the activation of the spatial position coordinate point $(x,y)$ in the last residual cell of channel $k$. With channel $k$, the result of the GAP that has been performed is denoted as $F_{k}$, and $F_{k}$ is $\frac{{1}}{H * W}\sum\nolimits_{x,y} {f_{k} (x,y)}$. Hence, given the category $c$, the result of classifier $S_{c}$ is:

1 $$S_{c} = \sum\limits_{k} {w_{k}^{c} } F_{k} = \sum\limits_{k} {w_{k}^{c} } \frac{1}{H * W}\sum\limits_{x,y} {f_{k} (x,y)} = \frac{1}{H * W}\sum\limits_{x,y} {\sum\limits_{k} {w_{k}^{c} } f_{k} (x,y)}$$

where $w_{k}^{c}$ represents the weight of the model for channel $k$ in the final dense layer corresponding to category $c$. It follows that $w_{k}^{c}$ is important for the final class judgment, and each position element in CAM is defined in category $c$ is:

2 $$CAM_{c} (x,y) = \sum\limits_{k} {w_{k}^{c} } f_{k} (x,y)$$

In the case where the input image is classified as target class $c$, the CAM identifies the significance of each location pixel $(x,y)$ on feature map's spatial grid. Up-sampling the CAM to match the size of the original input image, the most relevant region to the target category $c$ can be identified.

### G. Intermediate layer feature map output module

Applying the Hook technique to ResNet enables the extraction of feature output maps from the intermediate layers of the neural network, facilitating a deeper analysis of the network’s feature extraction process. The Hook function is applied by registering it on a specific layer or module within ResNet. This Hook function is a custom callback function. During the forward propagation of the input image through ResNet, the registered Hook function is triggered, capturing the output feature maps of the specified layer or module in accordance with the specified instructions. These feature maps can then be employed for subsequent analysis, visualization, or further processing.

### Informed consent

Informed consent was obtained from all subjects involved in the study.

## Results

In this section, all five proposed deep learning models are evaluated for their performance on a real pathology image set. Our experiments are structured into three parts: first, we conduct training and testing of these five deep learning models on the target dataset to identify the most effective model for vitiligo classification. Second, we analyze the visual interpretation to determine if the internal weighting parameters will provide valuable information for vitiligo diagnosis. Finally, we visualize the output feature maps for the intermediate layers of ResNet, Swin Transformer Base, and Swin Transformer Large. This visualization enables us to observe the response regions of neurons and the important features during the feature extraction process.

The classification performance was evaluated based on accuracy (ACC), sensitivity (SEN), specificity (SPE), precision (PRE), and F1-score with vitiligo considered as the positive example. These metrics were computed using True Positive (TP), True Negative (TN), False Positive (FP), and False Negative (FN) respectively. ACC serves as a measure of the overall correctness of predictions, irrespective of whether they pertain to positive or negative samples. It reflects the ratio of correct predictions to the total predictions made. SEN represents the proportion of tests that accurately detect true instances of disease, essentially capturing the true positive rate. On the other hand, SPE denotes the proportion of tests that accurately identify non-diseased individuals, constituting the true negative rate. PRE represents the proportion of samples with a positive prediction that is actually positive. F1-score is a weighted average of PRE and Recall. PRE reflects the model's ability to discriminate between negative samples, and the higher the PRE, the better the model's ability to distinguish between negative samples; Recall reflects the model's ability to recognize positive samples, the higher the Recall, the better the model's ability to recognize positive samples. The F1-score is a combination of the two, with higher F1-scores indicating a more robust model.

3 $$ACC = \frac{TP + TN}{{TP + FP + TN + FN}}$$

4 $$SEN = {\text{Re}} call = \frac{TP}{{TP + FN}}$$

5 $$SPE = \frac{TN}{{TN + FP}}$$

6 $$PRE = \frac{TP}{{TP + FP}}$$

7 $$F1{ - }score = {2} \times \frac{{\Pr ecision \times {\text{Re}} call}}{{\Pr ecision + {\text{Re}} call}}$$

In addition to these quantitative metrics, we generated receiver operating characteristic (ROC) curves for these five models. These curves are employed for a binary categorization task to distinguish between vitiligo and non-vitiligo skin diseases.

### A. Performance of five models

**Figure 5:**
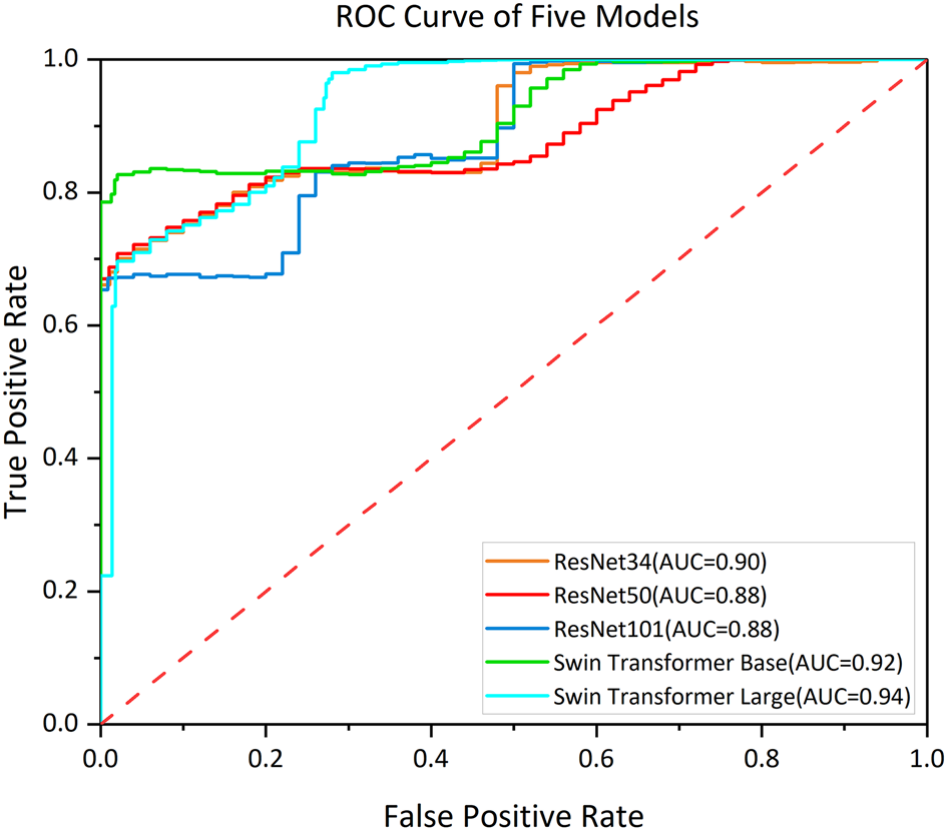
ROC curves of five models.

**Figure 6:**
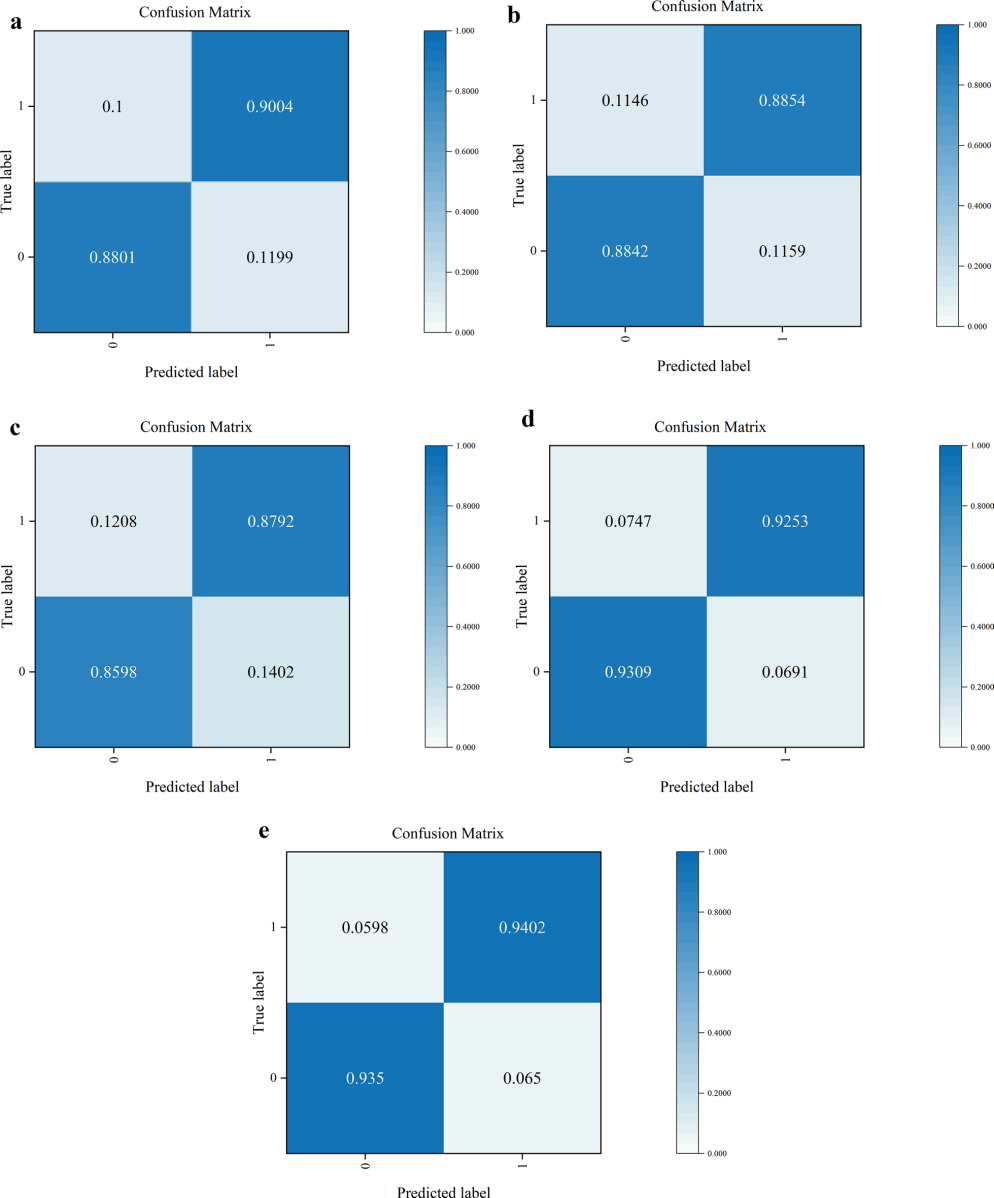
Confusion matrixes of five models on test set: (**a**) ResNet34; (**b**) ResNet50; (**c**) ResNet101; (**d**) Swin Transformer Base; (**e**) Swin Transformer Large.

The ROC curves of the five models used in this research were analyzed, and the final results of the test set are presented in Fig. 5. Notably, in the case of AUC, the Swin Transformer Large model outperforms the other models, achieving a value of 94%. AUC is a significant performance metric, indicating the reliability of prediction outcomes, especially for binary classifiers. Furthermore, to provide a more detailed assessment, a confusion matrix was utilized to quantify and visualize the performance of five models (Fig. 6). The rows and columns of the matrix correspond to the predicted and actual classes, respectively, where 1 represents vitiligo and 0 represents other skin diseases that are not vitiligo. It is noteworthy that the Swin Transformer Large model exhibited the highest sensitivity at 94.02%%, and also a commendable specificity at 93.5%. Conversely, the Swin Transformer Base model demonstrated a slight reduction in its ability to accurately classify both negative and positive samples, with specificity and sensitivity values of 93.09% and 92.53%, respectively. Among the networks in the ResNet series, ResNet34 emerged as the top performer with a classification accuracy of 89.26%. After assessing the performance of five models, it was evident that the Swin Transformer Large model had the highest accuracy of 93.82% (as indicated in Table 1). Consequently, among the five models, Swin Transformer Large stands out as the preferred choice for the diagnosis of vitiligo based on dermatoscopic images.

**Table 1:** Performance of five models on test set.

Models	ACC (%)	SEN (%)	SPE (%)	AUC	PRE (%)	F1-score (%)
ResNet34	89.26	90.04	88.01	0.90	92.46	91.23
ResNet50	88.49	88.54	88.41	0.88	92.58	90.51
ResNet101	87.18	87.92	85.98	0.88	91.10	89.48
Swin transformer base	92.74	92.53	93.09	0.92	95.62	93.75
Swin transformer large	93.82	94.02	93.50	0.94	95.93	94.97

Furthermore, to ensure easy replication and validation of the research methodology, we analyze the proposed methodology in comparison with some state-of-the-art (SOTA) methods that have performed well^29,30^. Considering that most of the recent advances in the use of dermoscopic images have been aimed at bridging the gap between clinical and dermoscopic images^31,32^. Both^29,30^ used datasets of clinical images.

The comparative analysis results, as presented in Table 2, reveal that the model introduced in this paper exhibits inferior performance when compared to the method proposed in^29^ concerning the public dataset. Notably, the observed maximum accuracy difference is approximately 4%. This discrepancy could be attributed to variations in dataset characteristics. Specifically, the public dataset predominantly comprises skin images from non-smooth regions such as arms, whereas the dermoscopic images utilized in our study predominantly feature smoother regions. This dissimilarity in dataset composition emerges as a potential contributing factor to the discerned algorithmic differences.

**Table 2:** Performance of proposed models and other SOTA methods on different dataset.

Dataset	Models	ACC	SEN	SPE	PRE	F1-score
Public dataset collected from seven public dermatology atlas websites: DermNet, DermNet NZ, AtlasDerm, DermIS, SD-260, Kaggle, and DanDerm	(a) Proposed models					
	ResNet34	84.43%	84.97%	83.60%	85.38%	85.17%
	ResNet50	85.32%	85.45%	85.19%	86.67%	86.06%
	ResNet101	86.82%	87.79%	85.71%	87.38%	87.58%
	Swin Transformer Base	90.80%	90.61%	91.01%	91.90%	91.25%
	Swin Transformer Large	92.78%	92.96%	92.59%	93.40%	93.18%
	(b) Reference^29^					
	VGG	96.77%	97.20%	96.30%	96.73%	96.80%
	ResNet	95.27%	95.10%	95.60%	96.19%	95.80%
	DenseNet	96.27%	96.20%	96.10%	96.70%	96.20%
Person dataset provided by Department of Cosmetic Laser Surgery in the Hospital for Skin Disease and Institute of Dermatology, Chinese Academy of Medical Sciences (CAMS) & Peking Union Medical College	(a) Proposed models					
	ResNet34	81.65%	82.04%	81.00%	87.72%	84.78%
	ResNet50	82.40%	82.63%	82.00%	88.46%	85.45%
	ResNet101	83.52%	84.43%	82.00%	88.68%	86.96%
	Swin Transformer Base	86.89%	87.43%	86.00%	91.82%	89.57%
	Swin Transformer Large	87.64%	88.02%	87.00%	91.88%	89.91%
	(b) Reference^30^					
	YOLO V3	85.02%	92.91%	72.00%	84.70%	88.62%

Moreover, it is noteworthy that the proposed method demonstrates a marginally superior performance on the personal dataset compared to the method in^30^. This nuanced improvement could be indicative of the adaptability and efficacy of our proposed approach in handling the specific attributes inherent to the personal dataset. Further analysis and exploration of these dataset-specific intricacies are warranted to comprehensively understand the observed performance variations between the proposed method and existing methods. In summation, our models obtain good results on several types of datasets, which validate the stability and generalization of the models.

### B. Interpretive visuals generated by the CAM module

**Figure 7:**
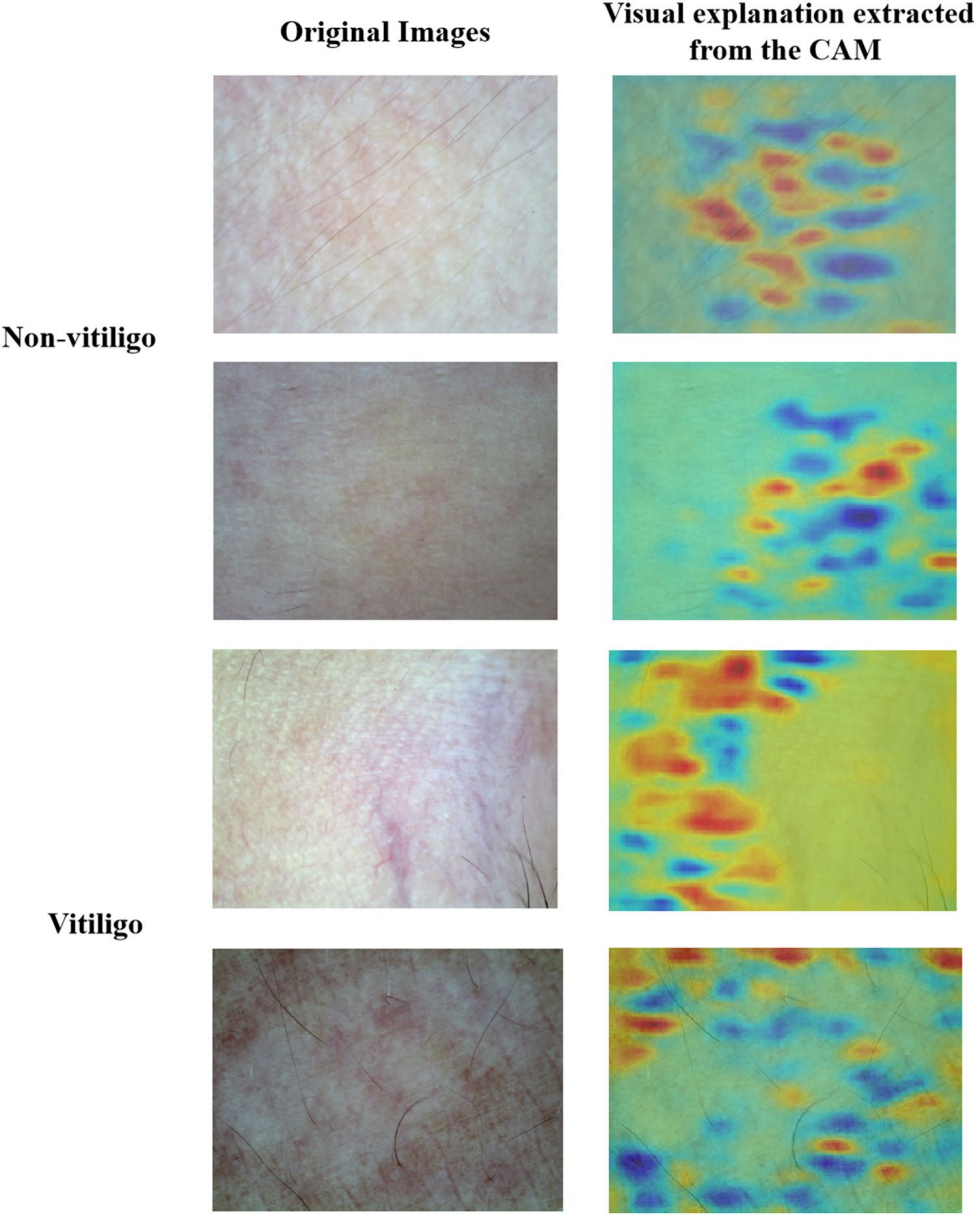
Examples of visual interpretation.

There are some instances of visual interpretation that are extracted from ResNet's CAM module (Fig. 7). The original images corresponding to these results are also included for further analysis. In these visualizations, red areas indicate regions where the network is activated, while blue areas signify regions with no activation. The darker red color indicates that the region has a higher contribution value to the model discrimination. Notably, the activation is concentrated in areas associated with skin lesions, from the figure, it can be seen that there are two bases of discrimination when the model makes judgments: one is based on whether the area of the white spots is large and continuous and combined with the edge characteristics of the lesion area, and the other is based on the color difference for differentiation, i.e., the difference in pigmentation between the lesion area and the other normal skin areas. According to the comprehensive analysis conducted by experienced dermatologists, the model captures the two bases and features of clinical diagnosis, i.e. edge and pigmentation, in the judgment. The interpretability of the class activation map cues is in great agreement with the physician’s clinical experience. This suggest that the CAM-generated heat maps are capable of highlighting category-specific areas of interest at critical diagnostic points. When analyzing the class activation maps, we observed that activations were not presented in all regions associated with vitiligo. The distribution density of activations did not exactly correspond to the features of vitiligo. This may due to the fact that the activation layers selected when generating the class activation maps may not provide enough information to accurately reflect the key features of vitiligo. Different levels of activation may highlight different image features. This results in failure to capture features associated with vitiligo at specific levels.

However, despite these limitations, we would like to acknowledge and emphasize the insights provided by class activation maps in terms of visual interpretation. By localizing key regions for classification, we can guide vitiligo diagnostic decisions. Although the distribution of activations may not exactly match the reality of the lesion, this analysis still provides us with information about which regions of the image the model is focusing on, thus providing a strong guide to diagnosis.

### C. Visual explanations from feature maps

**Figure 8:**
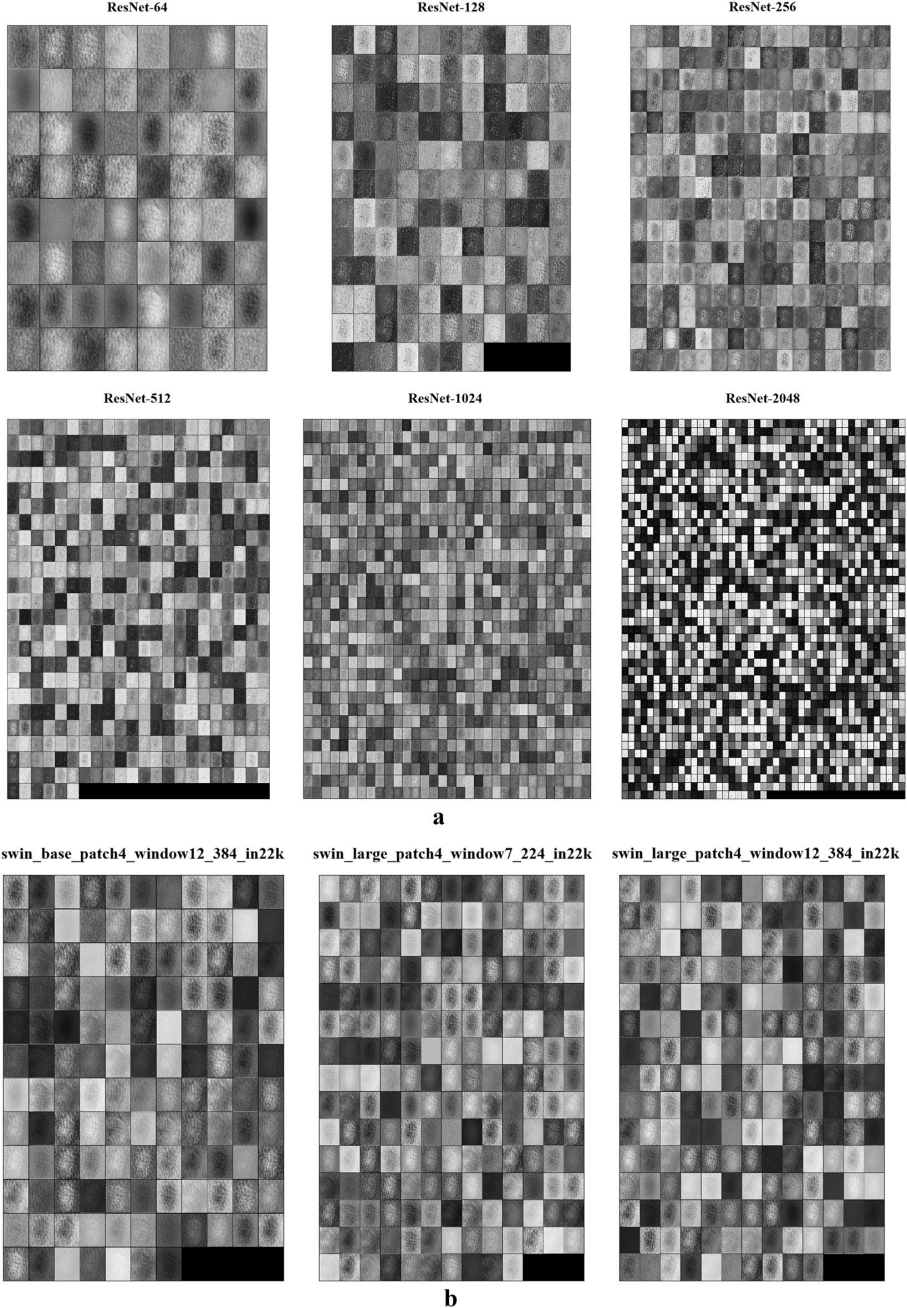
Examples of feature maps for different channels extracted from: (a) ResNet; (b) Swin Transformer.

By utilizing Hooks to access feature maps from the neural network's intermediate layer, the convolutional layers are intercepted to obtain feature representations of the different layers. These feature maps serve multiple purposes, including visual interpretation, analysis, and enhancing the interpretability of deep learning models. In Fig. 8a, the ResNet middle layer feature output image is displayed, with each grid in different layouts representing a feature map. In Fig. 8b, the feature layer output images of Swin Transformer Large and Swin Transformer Base are showcased. Due to the depth and complexity of the models, each convolutional layer extracts features at different levels and abstraction. As a result, the feature maps output from multiple intermediate layers can synthetically represent various visual information within the input image. This aids in better understanding the decision-making process and performance of the deep learning model.

### Institutional review board statement

Written informed consent for the use of identifying images was obtained from all patients. The study was approved by the Ethics Committee of the Fourth Military Medical University in accordance to the Declaration of Helsinki Principles.

## Discussion

Recently, there has been a notable surge in interest surrounding the application of deep learning in medical diagnostics. Particularly, deep learning has demonstrated exceptional capabilities in tasks associated with image classification, with its application extending into the field of dermatology^33^. Seung et al. proposed a classification of clinical images encompassing 12 skin diseases using a deep learning algorithm, achieving a final average classification accuracy of 90%^34^. Andre et al. employed CNNs for skin cancer classification, achieving results comparable to the expertise of all evaluated experts and demonstrating a similar level of competence as dermatologists^35^. However, this is only the effect observed in experimental studies. In real-world settings, the results of models compared to experts need to be revisited and explored^36^. Furthermore, several studies have demonstrated the remarkable diagnostic and classification abilities of deep learning in tasks related to melanoma image analysis^37–39^. Which further promotes the development of deep learning in the field of dermatology.

Up to now, only a limited number of studies have delved into the application of deep learning in the context of vitiligo, and most of them have relied on publicly available datasets^40^. For example, Guo, L. et al. developed and validated a hybrid artificial intelligence (AI) model utilizing deep learning for the objective measurement and color analysis of vitiligo lesions. The accuracy achieved in detecting vitiligo lesions using the YOLO v3 architecture was reported at 85.02%^41^. Another study proposed an effective intelligent classification system for vitiligo, which generated high-resolution vitiligo images under the wood lamp and demonstrated high precision in classifying these images, achieving a classification accuracy of 85.69%^42^. In comparison to other skin diseases, the research on extensively trained vitiligo image datasets and high-precision diagnostic systems is still in its early stages. In this study, an accurate diagnostic system with interpretable vitiligo dermoscopic images was developed based on a deep learning model.

Five deep learning models were selected for comparison in this study, primarily comprising two network structures ResNet and Swin Transformer, along with their variants, ResNet34, ResNet50, ResNet101, Swin Transformer Large, and Swin Transformer Base. It is noteworthy that Swin Transformer is a relatively new network. In previous studies, ResNets have been used widely for dermatological image segmentation and classification and have demonstrated excellent performance^43–45^. Swin Transformer is a deep learning model based on Transformer architecture. It has found extensive applications in the field of medical image processing and has achieved remarkable results in computer vision tasks. Consequently, we chose to investigate the performance of these two widely used and innovative deep learning networks in the specific context of vitiligo diagnosis. The experimental results indicate that among the ResNets, ResNet34 performs slightly better than ResNet50, while ResNet101 exhibits the least favorable results. Generally, with an increase in network depth, the performance of ResNet gradually improves, as deeper structures tend to capture finer details and features in images more effectively. However, it’s noteworthy that on specific datasets or tasks, ResNet34 may outperform ResNet50^46^, and ResNet34 with fewer parameters could also be more robust and better generalized, particularly on smaller datasets where the data size is limited. It’s important to acknowledge that performance comparisons are influenced by various factors, and different studies may reach slightly different conclusions^47^. Swin Transformer, proposed as a novel model for computer vision in 2021, has demonstrated wide applicability in various tasks, including image segmentation, restoration, and reconstruction^48–50^. Swin Transformer has been used in medical image processing through its hierarchical structure and self-attention mechanism, which demonstrates a robust capability for feature extraction and modeling. This presents promising opportunities for innovation in medical image analysis and diagnosis. Currently, there have been limited studies reporting the utilization of Swin Transformer in the context of medical images^51–53^. As far as we are aware, our study stands out as one of the relatively few instances where the Swin Transformer has been applied to the analysis of dermoscopic images. As the optimal model in our task, Swin Transformer achieved an accuracy of 93.82% and 92.74% for both specifications, respectively. This indicates the potential of Swin Transformer in medical applications, warranting further research and exploration in the field of medicine.

## Conclusion

Vitiligo is a common hypopigmentation disease, and its final diagnosis usually requires a combination of professional doctors' experience and test results from specialized instruments. With the help of computer vision and deep learning technology, it can provide an auxiliary means to help doctors diagnose vitiligo more accurately. The objective of this study is to evaluate the performance of multiple deep learning models using vitiligo image samples and subsequently identify five models with optimal diagnostic performance, including ResNet34, ResNet50, ResNet101, Swin Transformer Base, and Swin Transformer Large. These models were then utilized to develop a vitiligo diagnostic system that not only provides a disease label but also generates a visual diagnostic report displaying the possible regions associated with the disease. The outcomes produced by the CAM module effectively emphasize specific areas relevant to each class within diagnostic points, thereby assisting in decision-making during the diagnosis of skin conditions. Additionally, the use of feature output maps from the middle layer of the neural network enhances the understanding of how the model processes input images for tasks such as classification or localization. This integrated visual and informational output helps to improve the interpretability of the system, providing physicians with more comprehensive and in-depth insights that enhance confidence and decision-making during the diagnostic process.

The results of this study demonstrate that deep learning techniques have achieved significant accuracy gains in vitiligo diagnosis and provide comprehensive visual and informative outputs for diagnostic results. This not only emphasizes the excellence of deep learning models in vitiligo diagnosis but also suggests the potential value of their application in a broader diagnostic setting covering a wide range of dermatological conditions. This finding highlights the great potential of deep learning techniques in the field of dermatological imaging and also emphasizes the urgency of delving deeper into this area in the future. These encouraging results not only provide more reliable diagnostic support for patients with vitiligo but also lay a solid foundation for advancing the diffusion of deep learning techniques in real-world dermatologic diagnostic applications.

## Data Availability

The data presented in this study are available on request from the corresponding author. The data are not publicly available due to privacy or ethical concerns.
